# Mixed-effects location scale modeling of stress and contextual factors on overeating: a real-world observational study

**DOI:** 10.1038/s41366-025-01987-z

**Published:** 2026-01-20

**Authors:** Saki Amagai, Xingruo Zhang, Farzad Shahabi, Christopher Romano, Tammy Stump, Donald Hedeker, Nabil Alshurafa

**Affiliations:** 1 Department of Preventive Medicine, Northwestern University Feinberg School of Medicine, Chicago, IL USA; 2 Department of Public Health Sciences, University of Chicago, Chicago, IL USA; 3 Department of Dermatology, University of Utah, Salt Lake City, UT USA

**Keywords:** Lifestyle modification, Weight management

## Abstract

**Objective:**

The objective of our 14-day technology-supported free-living study was to assess how psychological, environmental, and social factors affect overeating among participants with obesity.

**Methods:**

We recruited 47 adults with obesity (BMI ≥ 30 kg/m^2^), who collectively logged 2004 meals, wore and used study devices for meal verification, and completed daily food recalls administered by dietitians. Participants reported on stress, affect, hunger, and meal contexts through Ecological Momentary Assessments (EMA). To explore the factors influencing caloric intake per meal, we employed a two-level mixed-effects location scale model, capturing both between-subject (BS) and within-subject (WS) factors based on the EMA data. This is a secondary analysis of the SenseWhy study, focusing on the association between stress and intake.

**Results:**

Our analysis identified six BS factors (e.g., stress, perception of overeating, restaurant food, later meals, pleasure-seeking meal) and ten WS factors (e.g., biological hunger, perceived overeating, uncontrolled eating, social eating, restaurant food, snacks) to be significantly associated with caloric intake. Notably, participants who were more stressed, on average, consumed more calories (0.74; *p* = 0.002) with high consistency (−0.7; *p* = 0.048) between individuals. When stressed and not at home, participants consumed less calories (−0.62; *p* = 0.0043).

**Conclusion:**

Conventional strategies for managing stress-related overeating fall short. Effectively addressing overeating requires an understanding of both psychological and contextual factors.

## Introduction

Obesity, caused primarily by overeating relative to need [1], is a major public health crisis globally. In the US, more than 40% of the population is considered to have obesity [2]. Having obesity elevates the risk of developing various life-threatening conditions, such as diabetes, heart disease, stroke, and cancer, in addition to detrimental psychosocial effects [3]. The global prevalence of obesity, along with other psychological influences such as stress, worsened following the COVID-19 pandemic [4, 5] and is projected to increase dramatically by 2045 [6].

While obesity is a multifaceted issue, stress has been increasingly recognized as a significant driver of overeating and obesity. Stress has been shown to increase overeating of ultra-processed foods, which are energy-dense and correlated with overeating and obesity [7, 8]. Proposed mechanisms of stress-induced eating causing obesity include (1) psychologic indices (increased drive to eat [9], disinhibited eating), resulting in higher ultra-processed food intake [10] and increased calorie intake, and (2) physiologic effects through the hypothalamic-pituitary-adrenal axis, resulting in higher adipose storage and impaired glucose metabolism [11].

The impact of stress on overeating, however, is not straightforward and is heavily influenced by various environmental, social, and psychological factors. This complexity underscores the need for nuanced research approaches that not only adjust for these confounding factors but also explore the potential interplay between stress and these variables. To delve deeper into this complexity, Ecological Momentary Assessment (EMA) serves as a valuable methodological tool. It enables participants to use mobile devices or other electronic devices in their natural environment to report on their stress levels and other behaviors, thoughts, and feelings, close to real-time when meals are consumed. By selectively incorporating EMA factors that are predictive of overeating, it allows for a more focused understanding of how stress, alongside other variables, contributes to overeating and obesity. Recent studies using daily diaries and EMA have begun to yield insights about the relationship between stress and eating. For instance, these studies have found that stress predicts increased food intake [12] and between-meal snacking [13].

However, most prior studies examining predictors of eating behavior predominantly only focus on between-subject effects—how these factors [14], on average, differ between individuals—without adequately addressing how they vary within an individual over time or across contexts (within-subject effects). This gap in understanding the consistency and variability of overeating behaviors limits our ability to design targeted, personalized interventions for overeating. To our knowledge, this is the first study to apply mixed effects regression models using EMA data in the context of obesity. A key advantage of these models is their ability to incorporate random subject effects, which account for individual-level influences on repeated outcomes, thereby distinguishing between- and within-subject factors. Disentangling the effects of these factors at both the between- and within-subject levels enhances our ability to design more tailored and personalized treatments that adapt to an individual’s specific context. Additionally, consistent overeating behavior may suggest the formation of a habit, which requires disruption to mitigate its impact [15, 16]. Recent interest in behavioral consistency has grown [17], particularly in the context of just-in-time adaptive (JITAI), where understanding which factors should be targeted to modulate variability in overeating is crucial for the success of timely interventions.

## Materials and methods

### Study design and participant characteristics

We recruited 65 adults with obesity (BMI ≥ 30 kg/m^2^) residing in the Chicago Metropolitan Area for a free-living observational study. Participants wore a comprehensive sensing suite, including a wearable camera to visually confirm behavior, a wrist-worn sensor to monitor heart rate and activity, and a specialized necklace to detect food intake [18]. In addition, a smartphone app was used to log food and complete EMAs of environmental, social, and psychological factors related to meals. For example, for stress EMAs, participants answered the item “*How stressed or anxious do you feel right now?*” on a 5-point Likert scale (1 = Not at all, 5 = Extremely) immediately before and after each meal. The pre-meal rating served as the primary indicator of acute perceived stress for the present analysis.

**Fig. 1:**
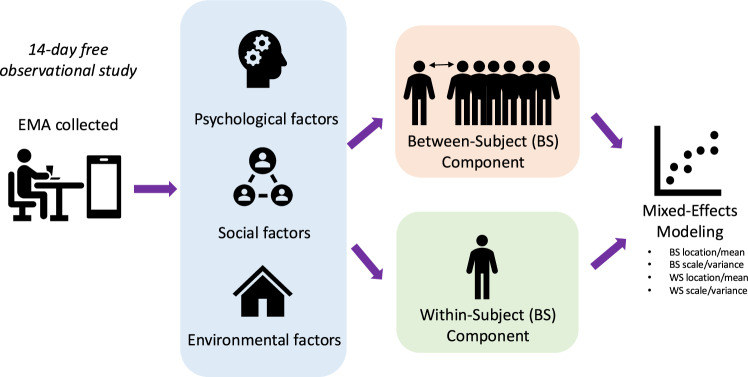
Analytical workflow overview. Overview of the analytical workflow. In our 14-day technology-supported free observational study, 47 adults with obesity completed their Ecological Momentary Assessments via a smartphone app to log their food and capture environmental, social, and psychological factors associated with the meals. These factors were then broken down into between-subject (BS) and within-subject (WS) components. We used a two-level mixed-effects location scale model to analyze both BS and WS components in caloric intake per meal.

To obtain a thorough account of dietary intake, a dietitian conducted 24-h dietary recalls via telephone using the five-step Automated Multiple Pass Method [19–21]. Both self-reported food logs, where participants documented their intake and photographed meals before and after consumption, and dietitian-administered recalls were utilized to ensure accurate reporting. This combined data allowed for precise calculation of caloric intake, which was the primary outcome measure. More details on the study design have been published [18]. The present manuscript reports secondary analyses, focused on the link between stress-related contextual factors and overeating (Fig. 1).

**Fig. 2:**
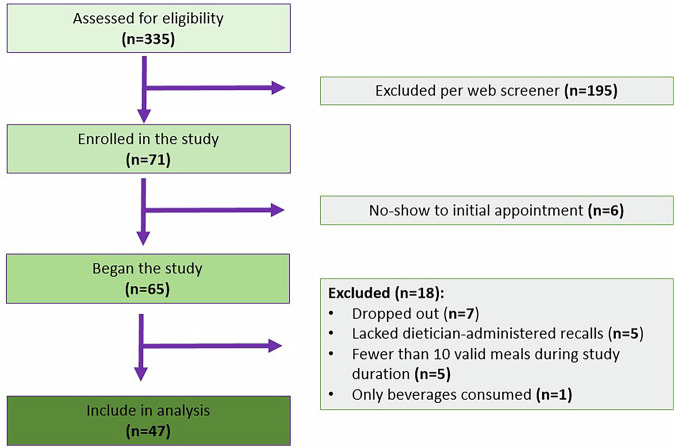
Participant selection. Out of 335 adults screened for eligibility, 71 enrolled in the study. Following adjustments for no-shows, 65 participants initiated the study. In the end, 47 participants, contributing a total of 2004 meal-level observations, were included in the final analysis. Participants were excluded if they dropped out, had missing dietary recall data, recorded fewer than 10 meals over the two-week period, or only consumed beverages during meals.

Of the 335 adults assessed for eligibility, 71 enrolled in the study. After accounting for no-shows, 65 participants began the study. Ultimately, 47 participants, with a total of 2004 meal-level observations, were included in the final analysis (Fig. 2). Participants were excluded if they dropped out, had missing dietary recalls, recorded fewer than 10 meals over the two-week period, or only consumed beverages during meals. Additional meal characteristics are detailed in Supplementary Table 1. As defined in our protocol, participants currently engaged in weight loss interventions or who had lost 15 or more pounds in the previous three months were excluded to focus on established eating habits. Full details of the study design have been previously published [18].

We obtained a total of 2004 meal-level observations from these 47 participants with obesity. Beverage-only meals were excluded. Features of meals collected are shown in Supplementary Table 1. As previously defined, participants were excluded from the study itself if they were currently dieting with the goal of losing weight or had lost 15 or more pounds in the previous three months [18].

This study was reviewed and approved by the National University Institutional Review Board (STU00204564), and consent was obtained from all participants. We have followed the STROBE (Strengthening the Reporting of Observational Studies in Epidemiology) guideline in writing this manuscript.

### Purpose

The primary aims of this study were to: 1) investigate the role of stress, alongside contextual factors (i.e., psychological, environmental, and social), in influencing caloric consumption, and 2) examine how stress, in combination with these additional factors, impacts the consistency and variability of calorie intake. By doing so, this study seeks to illuminate the critical role these factors play in overeating episodes.

### Statistical analysis

Mixed-effects location scale models [22], which allow for simultaneous modeling of both the outcome mean and within-subject variability as a function of covariates, to explore the psychological, environmental, and social factors contributing most to overeating within individuals. A detailed description of all factors considered in this study is provided in the appendix. These two-level models account for between-subject (BS) heterogeneity and within-subject (WS) dependence by incorporating multiple observations per participant. No violations of model assumptions were noted.

In this EMA study, participants logged a mean of 42.6 meals (range: 19–82 meals), enabling the application of an extended modeling approach that conventional mixed-effects models could not accommodate. Specifically, we modeled WS variances (referred to as scale) as a function of covariates, in addition to assessing the effects of covariates on overall mean levels (referred to as location). In the location model, we examined both the effects of BS components of covariates on mean caloric intake (e.g., did participants who are more stressed on average consume more than participants with less stress?) and the effects of WS components of covariates on the mean caloric intake (e.g., did each participant consume more when they were more stressed?) Similarly, in the scale model, we assessed both BS and WS components’ effects on WS variability in caloric intake. For time-varying, prompt-level covariates, we constructed BS components by calculating subject means, and WS components by subtracting the subject mean from the overall value, as outlined in Hofmann et al. [23] and Yaremych et al. [24]. To clarify, we labeled variables with either WS or BS to distinguish between the respective components. For example, StressBS reflects a participant’s mean stress level before meals, while StressWS, represents deviations from this mean at specific prompts.

Using these BS and WS components for time-varying covariates, we first conducted univariate analyses to identify which covariates to include in our final multivariable model Covariates were retained if either their BS or WS components were significant in either the location or scale model. In both the univariate and multivariate models, we controlled for sex, biological hunger, snacking (whether participants snacked or not), and restaurant variables (whether meals were eaten at a restaurant or via takeout), based on prior knowledge of their influence on overeating. These factors also showed significant effects on overeating in our analysis. We did not control for BMI, age, or race/ethnicity, as these variables were not significantly associated with the outcome.

For all analyses, our outcome variable—caloric intake (kcal) per meal—was modeled on a logarithmic scale to approximate normality. To ensure consistency across the different Likert scales used in the EMA reporting (Supplementary Table 1), we normalized the EMA responses to a 0–1 range. In addition to the univariate analyses, given the well-established role of stress in influencing calorie consumption, we examined the interaction effects between stress and other covariates in both the location and scale models (Supplementary Figure 1 and 2). If any interaction was significant in either part of the model, the interaction term was included in the final multivariable model.

### Definition of overeating

In the location model, overeating is defined as consuming more calories per meal than the participants’ average intake, with psychological, environmental, and social factors as key influencers. Similarly, in the scale model, overeating is characterized by caloric intake that exceeds an individual’s typical meal consumption, driven by these same contextual factors. This definition underscores the substantial role of contextual influences on increased caloric intake among individuals with obesity.

## Results

A total of 2004 meals consumed by 47 participants with obesity with a mean [SD] BMI of 37.6 [7.2] were used to build the mixed-effects location scale (MELS) model. The baseline characteristics of the participants are presented in Table 1 (18 [38.3%] Non-Hispanic Black, 16 [34.0%] Non-Hispanic White, 31 [27.7%] Other). A total of 36 (76.6%) participants were female with a mean age of 40.1 (range: 21–66). The meals consumed by the participants had a mean [SD] calorie intake of 520.8 kcal [396.6] (Supplementary Table 2).

**Table 1:** Sample characteristics.

Characteristic	*N* = *47*
Age, mean (SD)	40.1 (13.1)
BMI, mean (SD)	37.6 (7.3)
Sex, *n* (%)	
Female	36 (76.6)
Male	11 (23.4)
Race and ethnicity, *n* (%)	
Non-Hispanic Black	18 (38.3)
Non-Hispanic White	16 (34.0)
Other	13 (27.7)

Data are presented as *n* (%).

*BMI* body mass index.

### Univariate analysis and interactions

Supplementary Table 3 presents the results for the MELS models run separately for each covariate, illustrating their effects on both the location and scale of caloric intake. Based on these univariate analyses, statistically significant covariates were added to the multivariate analyses. Although stress was not significantly associated with caloric intake in the univariate analysis, we retained it in the final model due to its central importance in our hypothesis. Existing literature has consistently shown that eating behaviors shift under stress [25], and a prior research, including our own [26], demonstrated significant effects of stress on within-subject variability in caloric intake [24].

The univariate analyses revealed several noteworthy findings. Among between-subject (BS) components, participants who were generally more upbeat consumed fewer calories on average. Additionally, participants who tended to eat later in the day consumed more calories with greater variability (i.e., less consistency). For WS components, eating more uncontrollably than is typical for the participant was associated with greater caloric intake. Similarly, higher-than-usual perceptions of overeating, eating for pleasure, consuming meals later in the day, or being in a social setting were all associated with increased caloric intake.

From the analyses of the interactions between stress and other covariates, we found the three interactions to be significant: 1) between BS stress and WS perceived overeating in the location model 2) between WS stress and WS location (at home vs. not at home) in the location model, and 3) between WS stress and BS hedonic eating in the scale model (Supplementary Table 3).

### Multivariable analyses

**Fig. 3:**
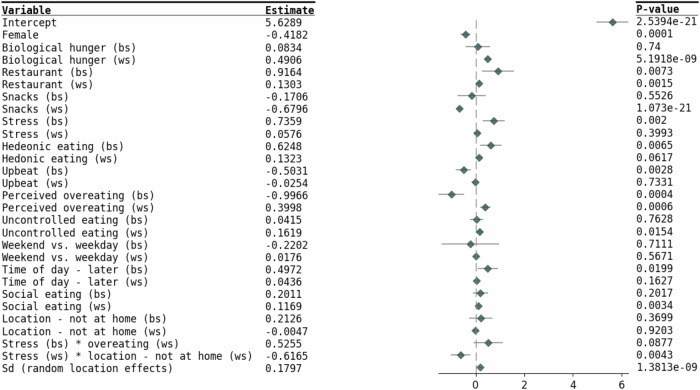
Forest plot for the location model. Estimates of location within the mixed-effects location scale models represent the outcome mean as a function of covariates. BS between-subject, WS within-subject.

**Fig. 4:**
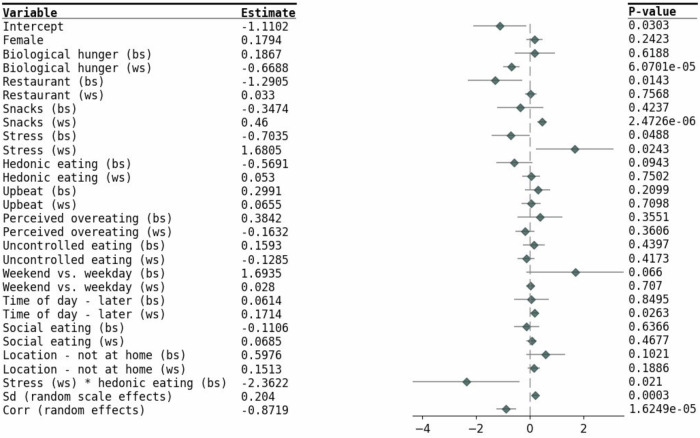
Forest plot for the scale model. Estimates of scale within the mixed-effects location scale models represent the outcome within-subject variability as a function of covariates. BS between-subject, WS within-subject.

Figures 3 and 4 present the results from the final model, which includes all the significant covariates from the univariate analysis, along with stress and its significant interactions terms. Among the BS components, participants who experienced higher average levels of stress consumed more calories, while those with a higher average perception of overeating consumed fewer calories. At an environmental and social level, participants who consumed more restaurant food/later meals or sought more pleasure by eating, on average, consumed more calories. In terms of WS variance, participants who consumed more restaurant food or reported higher levels of stress, on average, had lower variability in their caloric intake.

For WS components, psychological factors, such as increased biological hunger, perceived overeating, and uncontrolled eating that exceeded a participant’s usual behavior were associated with greater caloric intake. At the environmental and social level, eating in social settings or consuming restaurant food also led to higher caloric intake, whereas snacking, instead of having a full meal, was associated with lower caloric intake. Regarding WS variance, caloric intake was more variable when participants were stressed, while biological hunger led to less variability. Additionally, eating snacks or having meals later in the day was associated with greater variability in caloric intake.

As shown in Supplementary Figs. 1, 2, the effect of the WS component of stress varied based on the meal location and BS hedonic hunger levels. Specifically, when participants were stressed and outside their homes, they consumed significantly fewer calories (*p*-value = 0.0043). Increased stress was associated with more variability in caloric intake for individuals with low average hedonic hunger, while it was associated with reduced variability for those with high average hedonic hunger, although neither association was statistically significant.

## Discussion

In this 14-day free-living observational study of 47 adults with obesity, during which participants consumed 2004 meals, we demonstrated that higher momentary stress was associated with greater caloric intake and reduced meal-to-meal variability, even after adjusting for environmental, psychological, and social factors. When participants were stressed and outside their home, however, they logged fewer calories, suggesting that contextual factors may moderate stress-related overeating. Other psychological factors (e.g., greater biological hunger, perceived overeating, and feelings of lack of control in eating) were associated with increased calorie consumption relative to participants’ typical consumption. Environmental and social factors (e.g., restaurant food, social eating, later-day eating) were also associated with greater calorie intake. Together, these findings point to multiple, context-dependent influences on energy intake and underscore the potential value of interventions that address stress alongside situational cues (e.g., eating location) rather than treating stress as a single, uniform driver of overeating.

Our findings support the hypothesized links between stress and increased caloric consumption: participants who experienced higher levels of stress on average consumed more calories with greater consistency. This is consistent with previous studies showing that chronic stress is associated with unhealthy eating behaviors, such as choosing high-calorie, high-fat options, in both animal and human studies [9, 27]. Additionally, a prior study demonstrated that a stress-induced increase in cortisol hormone level was associated with greater food craving and intake [28].

We also found that when participants experienced acute stress at the occasion level, their caloric intake became more variable compared to when they were not stressed. This pattern is consistent with prior work showing that acute stress is associated with a higher likelihood of selecting energy-dense foods such as sweets or high-fat items [29, 30]. The simultaneous rise in average calories and calorie variability indicates that stressful moments coincide with the departures from an individual’s usual intake pattern. Importantly, these associations were heterogenous: a subset of participants recorded lower intake when feeling stressed or anxious in our study. Such variability highlights the importance of studying stress in context and accounting for person-level differences when assessing how momentary stress relates to eating behavior.

Stress-related shifts in eating appear to depend on both person-level traits and situational context. In our data, the association between momentary stress and calorie intake differed by hedonic-eating status. Although the simple-slopes tests were not statistically significant (see Supplementary Fig. 2), the point estimates suggest that participants classified as hedonic eaters tended to maintain lower meal-to-meal variability. In contrast, non-hedonic eaters displayed a wider range of caloric response under stress. These exploratory findings underscore the need to consider individual appetitive styles when examining how acute stress relates to eating behavior.

To our knowledge, this is the first free-living study to show an association between acute stress and lower caloric intake when eating occurred outside the home versus inside the home. One plausible explanation is reduced access to energy-dense foods in out-of-home settings, where the immediate convenience of a kitchen and pantry is absent and obtaining highly palatable items requires additional effort. This observation aligns with work suggesting that greater environmental barriers to food are linked to lower consumption. These findings tentatively support strategies that increase effort costs, such as limiting availability of high calorie snacks during stressful moments. Alternatively, reducing boredom [31] or increasing physical activity, spending time outdoors could help lower calorie consumption in stressful situations [32].

Mindfulness-based interventions aimed at reducing stress have shown promise in mitigating stress-related overeating. For example, in a recent study on pregnant women with obesity, participants in mindfulness strategies were associated with reductions in stress, depression, and emotional and external eating (i.e., eating for reasons other than hunger or caloric need) [33]. Digital platforms may extend these benefits by offering just-in-time adaptive interventions (JITAI) tailored to a participant’s emotional state in real time. In one study, Smyth and Heron evaluated whether JITAI stress-management reminders, sent to participants during moments of high stress or negative affect, were associated with reduced stress severity and the frequency of stressful events compared to random, untailored reminders [34]. Their findings suggest that individuals receiving tailored JITAI reminders experienced fewer stressful events, lower stress severity, and engaged in less frequent eating than those who received no reminders or only random reminders.

Beyond stress, several other factors were significantly associated with caloric consumption. Participants who ate more frequently at restaurants or had takeout were consistently associated with higher caloric consumption on average. Even at the occasion-level, meals from restaurants were associated with higher caloric intake. Providing clearer nutrition information in food outlets and wider access to nutrition education may assist consumers in making lower-calorie selections, although these strategies require prospective evaluation. Meals classified as being eaten “for pleasure” were likewise associated with higher calorie content, a finding that aligns with the typically energy-dense nature of indulgent foods. Higher self-reported biological hunger was also associated with greater caloric intake.

Indicators of perceived overeating displayed a split pattern. Across individuals, those who *frequently* felt they overate recorded slightly lower mean calories—possibly reflecting heightened awareness and compensatory restraint. Conversely, at the occasion level, participants who felt they were overeating consumed more calories during those moments. Taken together, these observations suggest that situational cues (e.g., holiday gatherings [35], restaurant settings [36]) and appetitive states (pleasure eating, hunger) are important contextual features to consider when designing interventions. Approaches worth testing include timely dietary reminders during holiday seasons, point-of-purchase calorie labeling, and digital self-monitoring tools that prompt reflection on portion size at the moment of choice.

This study has limitations. First, we did not examine the nutritional composition of the foods that were consumed, such as macronutrient breakdown or levels of fat, sugar, or processed content. It’s important to note that meals with higher caloric intake are not necessarily unhealthy. In future studies, exploring the relationship between stress, calorie intake, and the type or quality of food consumed would provide valuable insight. Second, we did not account for net caloric balance, which would require not only analyzing food intake but also considering energy expenditure, hormone profiles, and sleep patterns. Taking these factors into account would provide a more holistic understanding of how stress and other factors contribute to eating behaviors and overall health. Finally, we did not directly examine meal frequency or the distribution of eating occasions across the day, which could influence the associations observed and warrant investigation in future work.

## Conclusion

In summary, our study highlights the importance of psychological stress as well as various psychological, social, and environmental factors linked to the high-calorie food consumption. Stress alone offered limited explanatory power – overeating is a sophisticated problem that requires a multifaceted approach to intervention. Beyond stress, factors such as hunger and eating for pleasure were strongly associated with increased caloric consumption. Understanding the interplays of these factors in free-living settings is crucial, as it provides insight into real-world behavioral patterns outside of controlled laboratory environments, where much of the existing research has taken place. Moreover, we should consider going beyond single proximal determinants of overeating, such as stress or hedonic eating, and begin to understand how they co-occur with other social/environmental, allowing us to build interventions with decision rules around the right time and context to intervene.

## Supplementary information

Supplementary Table 1

Supplementary Table 2

Supplementary Table 3

Supplemental Figure 1

Supplemental Figure 2

## Data Availability

The data underlying this study are not publicly available because they contain sensitive participant information and are subject to Institutional Review Board (IRB) protections. Researchers may request access to the data on a case-by-case basis, contingent upon obtaining appropriate ethical approvals and completing necessary data-sharing agreements.

## Code availability

Analysis code available at https://github.com/amagais/sensewhy_mel.
