# Supplementary material for: Mixed-effects location scale modeling of stress and contextual factors on overeating: a real-world observational study
Source: Int J Obes (Lond). 2026 Jan 20;50(3):633–9. doi: 10.1038/s41366-025-01987-z (PMC12965874; doi:10.1038/s41366-025-01987-z)
Supplement: Supplementary file 1 — Supplementary Table 1 [file 41366_2025_1987_MOESM1_ESM.docx]

**Supplementary Table 1.** *Features of meals collected by study measures in the study*

| **Features** | **Category** | **Description** | **Type of variable** |
| --- | --- | --- | --- |
| Biological Hunger | Psychological | Degree to which hunger satisfaction motivates upcoming meal | Continuous  (1-5 point Likert scale) |
| Hedonic Eating | Psychological | Degree to which pleasure seeking motivates upcoming meal | Continuous  (1-5 point Likert scale) |
| Stress | Psychological | Perceived stress before and after a meal | Continuous  (1-5 point Likert scale) |
| Calmness | Psychological | Degree of feeling calm and peaceful | Continuous  (1-5 point Likert scale) |
| Upbeat | Psychological | Degree of feeling upbeat or excited | Continuous  (1-5 point Likert scale) |
| Loneliness | Psychological | Degree of feeling down or lonely | Continuous  (1-5 point Likert scale) |
| Uncontrolled Eating | Psychological | When I started eating, I just couldn’t seem to stop | Continuous  (1-4 point Likert scale) |
| Cognitive Restraint | Psychological | I consciously held back on how much I ate to keep from gaining weight | Continuous  (1-4 point Likert scale) |
| Perceived Overeating | Psychological | Did you eat more than you intended to? | Continuous  (1-5 point Likert scale) |
| Snacks | Behavioral | Snacks vs Non-Snacks | Binary |
| Restaurant | Environmental/Social | Food is made at a restaurant (dine in and take out) | Binary |
| Cooked meal | Behavioral | Food is cooked by participant (made from scratch, frozen, or prepackaged meals) | Binary |
| Social Eating | Environmental/Social | Eating alone, eating with others | Binary |
| Screen time | Environmental | Eating in presence of screen TV, computer, phone, etc.) | Binary |
| Time of day | Behavioral | Time meal  (Earlier = between 3am and 5pm; Later = between 5pm and 3am) | Binary |
| Weekend vs. Weekday | Behavioral | Meal consumed on weekday (0);  Meal consumed on weekend (1) | Binary |
| Location | Environmental/Social | At home (0); Not at home (1) | Binary |

Psychological, behavioral, and environmental/social features assessed at each meal using Ecological Momentary Assessment (EMA) and study measures. The table lists each variable, its category, description, and coding (continuous Likert-scale ratings or binary indicators) used as predictors of caloric intake in the mixed-effects location scale models.
