# Supplementary material for: Mixed-effects location scale modeling of stress and contextual factors on overeating: a real-world observational study
Source: Int J Obes (Lond). 2026 Jan 20;50(3):633–9. doi: 10.1038/s41366-025-01987-z (PMC12965874; doi:10.1038/s41366-025-01987-z)
Supplement: Supplementary file 2 — Supplementary Table 2 [file 41366_2025_1987_MOESM2_ESM.docx]

**Supplementary Table 2. Summary characteristics of the meals**

|  |  | Overall |
| --- | --- | --- |
| n |  | 2004 |
| Energy in kcal, mean (SD) |  | 520.8 (396.6) |
| Weekend vs. Weekday, n (%) | **Weekday** | 1417 (70.7) |
|  | **Weekend** | 587 (29.3) |
| Start Mealtime, mean (SD) |  | 14.8 (4.7) |
| Social Eating, n (%) | **Eating Alone** | 1432 (71.5) |
|  | **Eating with others** | 572 (28.5) |
| Other activity, n (%) | **Driving** | 59 (2.9) |
|  | **Nothing else** | 401 (20.0) |
|  | **Socializing** | 423 (21.1) |
|  | **Watching TV** | 764 (38.1) |
|  | **Work** | 357 (17.8) |
| Stress, mean (SD) |  | 0.2 (0.3) |
| Hedonic Eating mean (SD) |  | 0.7 (0.3) |
| Calmness, mean (SD) |  | 0.6 (0.3) |
| Upbeat, mean (SD) |  | 0.4 (0.3) |
| Loneliness, mean (SD) |  | 0.2 (0.3) |
| Biological Hunger, mean (SD) |  | 0.7 (0.3) |
| Cognitive Restraint, mean (SD) |  | 0.1 (0.2) |
| Perceived Overeating mean (SD) |  | 0.2 (0.3) |
| Uncontrolled Eating, mean (SD) |  | 0.3 (0.4) |
| Earlier vs. Later, n (%) | **Earlier** | 1168 (58.3) |
|  | **Later** | 836 (41.7) |
| Food Source n (%) | **Restaurant** | 425 (21.2) |
|  | **Cooked Meal** | 864 (43.1) |
|  | **Snacks/Cereal** | 715 (35.7) |

Summary characteristics of the 2004 meals logged during the 14-day study, including energy intake, timing, social context, concurrent activities, and meal source. Psychological variables (e.g., stress, hedonic eating, calmness, affect, hunger, restraint, perceived overeating, uncontrolled eating) are presented as mean (SD) on normalized 0–1 scales.
