# Supplementary material for: Mixed-effects location scale modeling of stress and contextual factors on overeating: a real-world observational study
Source: Int J Obes (Lond). 2026 Jan 20;50(3):633–9. doi: 10.1038/s41366-025-01987-z (PMC12965874; doi:10.1038/s41366-025-01987-z)
Supplement: Supplementary file 3 — Supplementary Table 3 [file 41366_2025_1987_MOESM3_ESM.docx]

**Supplementary Table 3.** Results from univariate analysis

|  | **BS Estimate (95% CI)** | **BS Estimate**  **(95% CI)** | **WS Estimate (95% CI)** | **WS Estimate**  **(95% CI)** |
| --- | --- | --- | --- | --- |
| **Stress** | 0.32  (-0.14, 0.78) | -0.22  (-0.77, 0.33) | 0.04  (-0.10, 0.17) | 0.08  (-0.24, 0.41) |
| **Hedonic Eating** | 0.12  (-0.42, 0.66) | -0.27  (-0.92, 0.35) | 0.27  (0.13, 0.41)* | 0.01  (-0.30, 0.32) |
| **Calmness** | -0.41  (-0.85, 0.04) | 0.24  (-0.29, 0.77) | 0.04  (-0.11, 0.18) | 0.08  (-0.26, 0.43) |
| **Upbeat** | -0.53  (-0.89, -0.16)* | 0.03  (-0.42, 0.48) | 0.08  (-0.06, 0.22) | 0.05  (-0.28, 0.39) |
| **Loneliness** | 0.38  (-0.02, 0.78) | -0.07  (-0.57, 0.43) | -0.09  (-0.26, 0.06) | 0.12  (-0.25, 0.48) |
| **Cognitive restraint** | -0.7  (-0.53, 0.39) | 0.28  (-0.84, 0.28) | -0.04  (-0.53, -0.38) | -0.25  (-0.66, -0.15) |
| **Perceived Overeating** | -0.33  (-0.86, 0.21) | 0.06  (-0.56, 0.69)* | 0.07  (-0.57, -0.69) | -0.20 (-0.52, -0.11) |
| **Uncontrolled eating** | 0.10 (-0.23, 0.43) | 0.12  (-0.25, 0.49) | 0.38  (0.26, 0.50)* | -0.14  (-0.43, -0.14) |
| **Weekend** | -0.68 (-2.3, 0.89) | 1.85  (0.07, 3.63)* | 0.01  (-0.05, -0.07) | 0.01  (-0.14, 0.16) |
| **Not Home** | 0.44  (-0.13, 1.02) | 0.39 (-0.30, 1.07) | -0.05 (-0.10, -0.09) | 0.10  (-0.11, 0.32) |
| **Later** | 0.56  (0.04,1.08)* | 0.29  (-0.36, 0.93) | 0.11  (0.046, 0.17)* | 0.17  (0.02, 0.31)* |
| **Social Eating** | 0.24  (-0.14, 0.62) | 0.13  (-0.32, 0.59) | 0.18  (0.10, 0.26)* | 0.09  (-0.09, 0.27) |

Univariate mixed-effects location scale model results for each psychological, behavioral, and contextual covariate, showing between-subject (BS) and within-subject (WS) estimates for location (mean caloric intake) and scale (within-subject variability). Estimates are presented with 95% confidence intervals; *p < 0.05.
