# Supplementary figures and images for: Mixed-effects location scale modeling of stress and contextual factors on overeating: a real-world observational study
Source: Int J Obes (Lond). 2026 Jan 20;50(3):633–9. doi: 10.1038/s41366-025-01987-z (PMC12965874; doi:10.1038/s41366-025-01987-z)

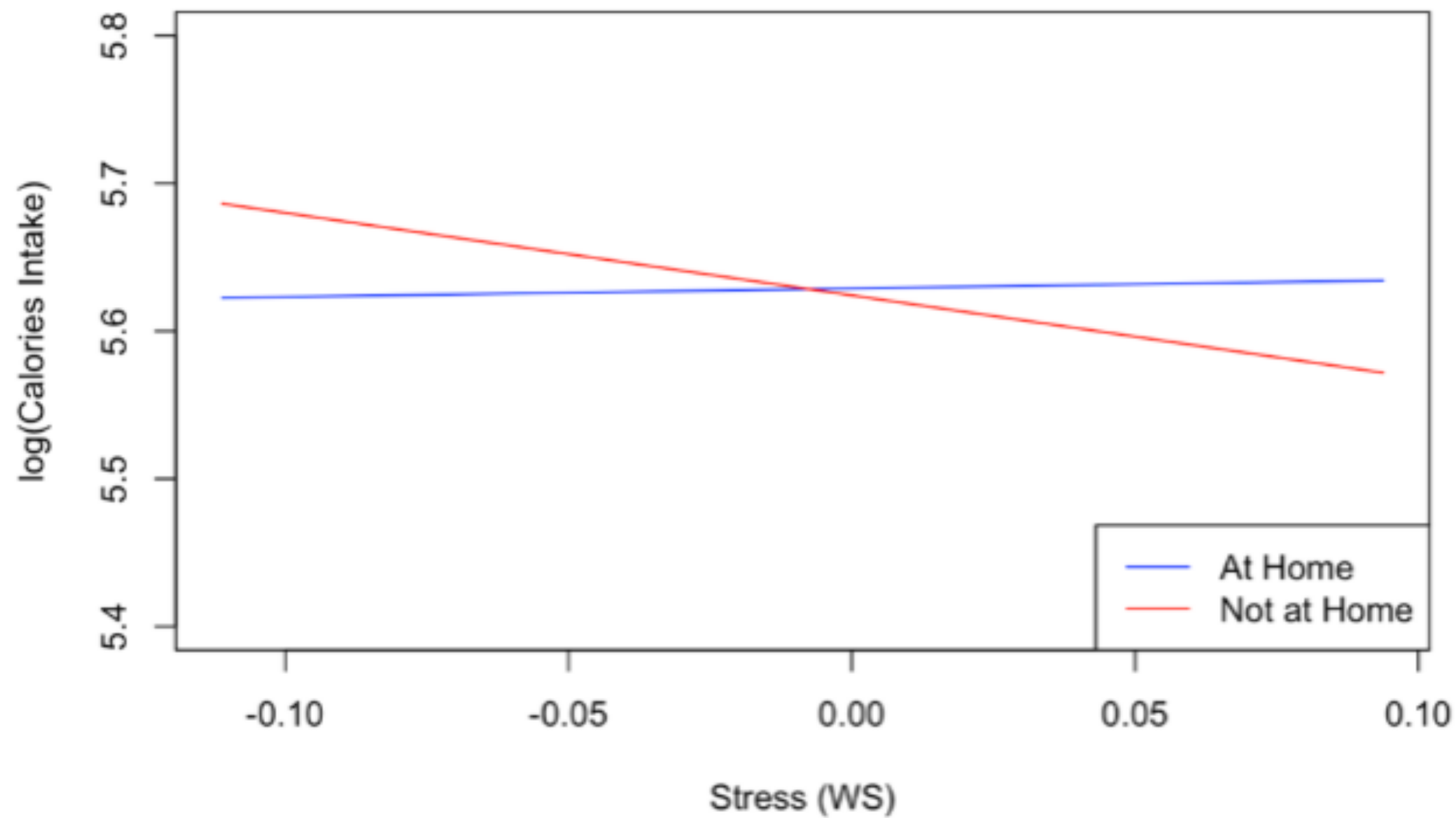

Supplement: Supplementary file 4 — Supplemental Figure 1 [file 41366_2025_1987_MOESM4_ESM.pdf]

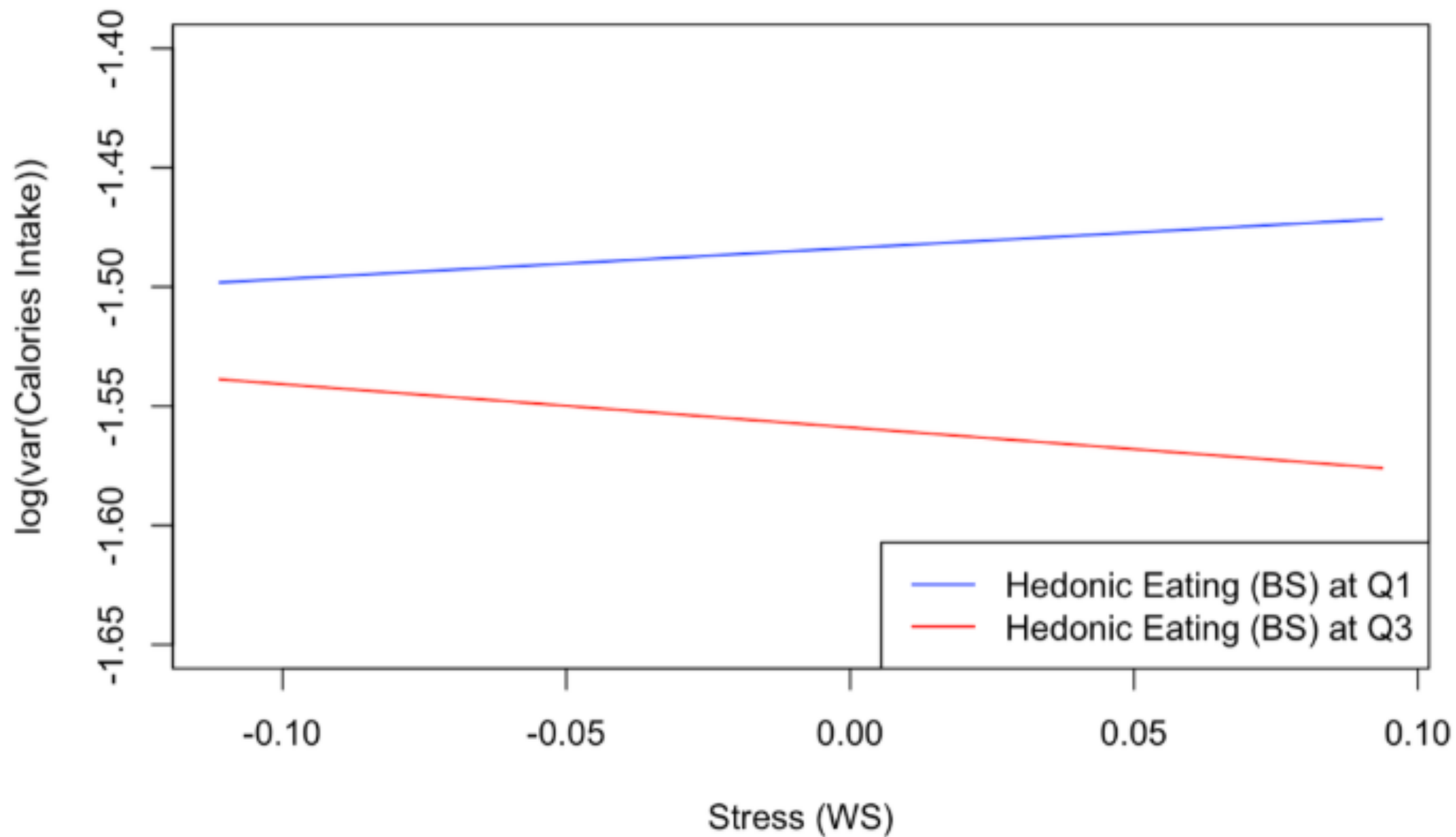

Supplement: Supplementary file 5 — Supplemental Figure 2 [file 41366_2025_1987_MOESM5_ESM.pdf]
